# Global Navigation Satellite System Receiver Positioning in Harsh Environments via Clock Bias Prediction by Empirical Mode Decomposition and Back Propagation Neural Network Method

**DOI:** 10.3390/s24072342

**Published:** 2024-04-07

**Authors:** Libin Du, Hao Chen, Yibo Yuan, Longjiang Song, Xiangqian Meng

**Affiliations:** 1 College of Ocean Science and Engineering, Shandong University of Science and Technology, Qingdao 266590, China; dulibin@sdust.edu.cn (L.D.); 18865940929@163.com (H.C.); lidarmeng@sdust.edu.cn (X.M.); 2 Space Star Technology Co., Ltd., Beijing 100086, China; songlj20220101@163.com

**Keywords:** receiver clock bias forecasting, random error, clock bias auxiliary positioning algorithm, satellite navigation

## Abstract

This paper proposes a novel method to improve the clock bias short-term prediction accuracy of navigation receivers then solve the problem of low positioning accuracy when the satellite signal quality deteriorates. Considering that the clock bias of a navigation receiver is equivalent to a virtual satellite, the predicted value of clock bias is used to assist navigation receivers in positioning. Consequently, a combined prediction method for navigation receiver clock bias based on Empirical Mode Decomposition (EMD) and Back Propagation Neural Network (BPNN) analysis theory is demonstrated. In view of systematic errors and random errors in the clock bias data from navigation receivers, the EMD method is used to decompose the clock bias data; then, the BPNN prediction method is used to establish a high-precision clock bias prediction model; finally, based on the clock bias prediction value, the three-dimensional positioning of the navigation receiver is realized by expanding the observation equation. The experimental results show that the proposed model is suitable for clock bias time series prediction and providing three-dimensional positioning information meets the requirements of navigation application in the harsh environment of only three satellites.

## 1. Introduction

For a traditional Global Navigation Satellite System (GNSS), the navigation receiver needs at least four satellites to perform three-dimensional positioning [1,2,3]. However, GNSS satellite signals are significantly impacted by the environment. In challenging environments such as densely populated urban areas with tall buildings, dense forests, and mountains, standard GNSS receivers often struggle to acquire signals from at least four satellites. Consequently, traditional positioning algorithms fail to perform three-dimensional positioning. This limitation severely disrupts the consistency of the positioning service and causes inconvenience [4,5,6]. Therefore, exploring solutions for GNSS receiver positioning in harsh environments holds paramount practical significance. It ensures the continuity of the positioning service and expands the range of applications for GNSS receivers [7].

In order to improve the continuity of GNSS receiver positioning in harsh environments, it is usually necessary to add additional hardware devices for external assistance [8,9], such as a combined navigation system [10], pseudo-satellite [11], vision measurement system [12], cellular signals [13], or barometric altitude measurement [14]. However, these auxiliary methods increase the hardware cost of the positioning system. When the GNSS receiver is positioned normally, in addition to solving the three-dimensional coordinates of the receiver, it can also provide the clock bias data from the receiver. As long as the navigation receiver’s clock frequency is usually stable and accurate, a prediction model can be built using historical clock bias data to forecast the clock bias for a period of time. In view of this, the GNSS receiver clock bias can be treated as a virtual satellite; the positioning process can be accomplished according to the clock bias prediction value. Compared with other methods, the clock bias predicting auxiliary method is an internal auxiliary method, with no need for additional hardware equipment. By simply embedding the demonstrated algorithm in the navigation receiver, it is possible to implement high-accuracy positioning in harsh environments. The application of the proposed method improves the adaptability of GNSS receivers and in the meantime decreases hardware complexity [15,16].

Due to the influence of elements such as the crystal oscillator quality of the GNSS receiver clock, the stability of the GNSS receiving signal transmission channel, and the GNSS satellite coordinate error, the actual GNSS receiver clock bias time series has obvious non-linear characteristics [17]. There are many methods that can be applied to such non-linear and unsmooth time series modeling. The effectiveness and robustness of traditional modeling and forecast methods such as fitting and extrapolation, the Grey System Model (GM) [18], and Auto Regressive and Moving Average (ARMA) [19] have been proven. With the recent developments in machine learning, the application of machine learning algorithms to GNSS signal processing has received increasing attention [20]. For better positioning accuracy, the Long Short-Term Memory neural network (LSTM) as a supervised learning model was leveraged for clock bias prediction during GNSS signal outages [21,22]. Liang et al. applied the Non-linear Auto-Regressive model with exogenous input (NARX) recurrent neural network to GNSS clock bias prediction, outperforming three traditional models, which are the Quadratic Polynomial model (QP), GM, and Autoregressive Integrated Moving Average model (ARIMA) [23]. To improve precision point positioning (PPP) accuracy, Liao et al. introduced the famous open source time series forecast model Prophet which was proposed by the Facebook company for clock bias prediction [24]. Based on the combination of Mind Evolutionary Algorithm (MEA) optimization and the Back Propagation Neural Network (BPNN) algorithm, Bai et al. proposed a MEA-BP clock prediction model with strong anti-interference ability [25]. Considering the characteristics and limitations of different models, a combination analysis and prediction model based on machine learning is an attractive choice.

In view of the non-linear characteristics of the GNSS receiver clock bias time series, with the help of time series analysis theory, this paper demonstrates a novel clock bias prediction model. The clock bias time series are decomposed into different components by the Empirical Mode Decomposition (EMD) method; then, each of the components is modeled and predicted according to their different characteristics using the BPNN method. The specific detailed implementation process of the proposed algorithm is elaborated and several verification field tests are carried out. The field test result shows that the proposed algorithm performs illustriously in navigation receiver clock bias time series predicting and could provide new insights into navigation positioning implementation in harsh environments with only three satellites.

## 2. Theory and Algorithm Details

According to the traditional theory of time series analysis and decomposition, the GNSS receiver clock bias series contain three parts: the trend item, period item, and random item. Therefore, the corresponding formulas of clock bias can be expressed as follows:

(1) $$\begin{array}{c} b\left(t\right)=c\left(t\right)+p\left(t\right)+r\left(t\right),t\in \left[1,n\right] \end{array}$$

In the formula, c(t) represents the trend item, which reflects the slow change in the clock bias series over a long period of time. p(t) represents the period item, reflecting the similarity in the clock bias series after a period of time. r(t) represents random items, reflecting the impact of other random factors.

Our EMD-BPNN GNSS receiver clock bias prediction model chooses a strategy that simplifies the time series analysis problem by decomposing to several parts and handling them separately. The EMD method is firstly used to decompose the clock bias time series and extract features like stable elements and residuals; then, the BPNN is established to learn and forecast the decomposed items. In the end, the predicted components are superimposed together to obtain the final GNSS receiver clock bias prediction result.

Due to the advantages such as intuitive analysis, adaptively basis function, and so on, the EMD method is usually used to analyze non-linear and non-stationary signals. The EMD method identifies and extracts signal features associated with various intrinsic time scales of the signal starting from finer temporal scales to coarser ones which makes it appropriate for the short-term prediction of navigation receiver clock bias series. With the help of the EMD method, the original navigation receiver clock bias data are decomposed into limited Intrinsic Mode Functions (IMFs) and residual quantity (RES) according to their own characteristics. For an input clock bias time series X(t), the detailed decomposition procedure is listed as follows:

Step 1: Find all the extremes of time series X(t), and use cubic spline interpolation to fit upper envelop $e_{1}\left(t\right)$ and lower envelop $e_{2}\left(t\right)$ of the original signal. The average value of them can be defined as follows:

(2) $$\begin{array}{c} e\left(t\right)=\frac{e_{1}\left(t\right)+e_{2}\left(t\right)}{2} \end{array}$$

Obtain c(t) by subtracting Equation (2) from the original signal:

(3) $$\begin{array}{c} c\left(t\right)=X\left(t\right)-e\left(t\right) \end{array}$$

Step 2: Check that c(t) satisfies the zero-mean IMF condition. If it does, treat c(t) as the first IMF and denote it as $f_{1}\left(t\right)$. If not, treat c(t) as a new original signal and repeat step 1.

Step 3: Separate $f_{1}\left(t\right)$ from X(t) and then use the remainder $RES_{1}\left(t\right)=X\left(t\right)-f_{1}\left(t\right)$ as the new original signal. Repeat the above steps to obtain *n* IMF components and stop the decomposition until the final residual becomes a monotone function. The original signal X(t) can be reconstructed from *n* IMF components and the final residual:

(4) $$\begin{array}{c} X\left(t\right)=\sum_{i=1}^{n}f_{i}\left(t\right)-RES_{n}\left(t\right) \end{array}$$

The GNSS receiver clock bias signal is a typically non-linear and non-stationary signal; it is decomposed into several IMF components each of which is stationary making future prediction possible; what is more, the EMD procedure also makes the future extraction much easier because the characteristics become more obvious after decomposition.

**Figure 1:**
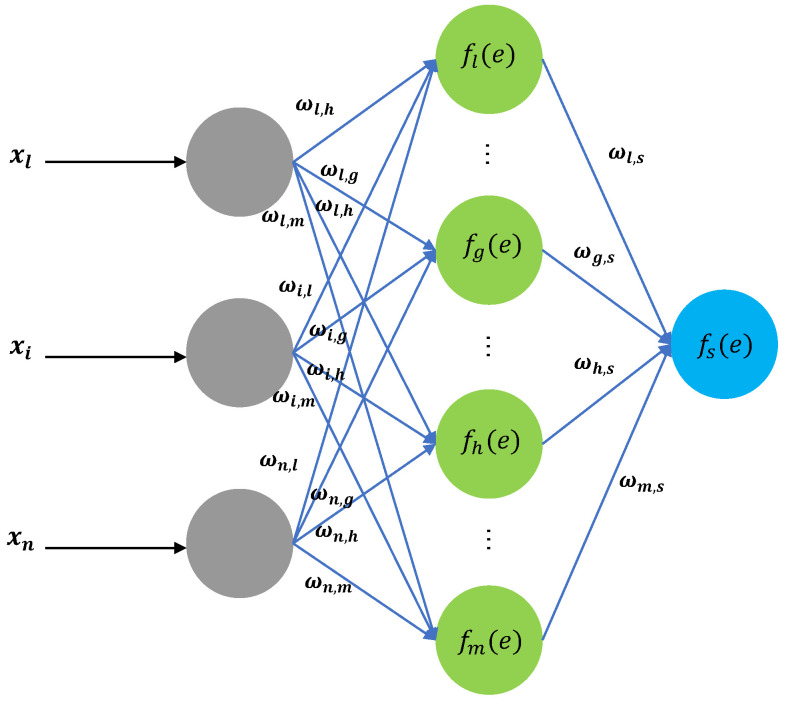
Typical schematic diagram of a BPNN structure.

The BPNN algorithm, which can adapt networks parameters such as weights and thresholds according to input training data, is utilized to forecast the decomposed IMF components. Therefore, by approximating a non-linear system after learning and training, it can give precise prediction to intricate non-linear signals like GNSS receiver clock bias time series. BPNN comprises three layers: the input layer, hidden layer, and output layer; a typical schematic diagram of a BPNN structure is shown in Figure 1; the notation pattern indicates the network connectivity. The hidden layer serves as the conduit for conveying vital information between the input and output layers. The iteration training process primarily involves two steps: information forward propagation and error backpropagation.

The basic unit of a BPNN is a neuron; the activation function of a neuron can be defined as follows:

(5) $$\begin{array}{c} y=f\left(\sum_{i=1}^{n}\omega_{i}x_{i}-\theta \right) \end{array}$$

In Equation (5), θ is the threshold, $\omega_{i}$ is the weight of the i-th neuron, and *n* is the total number of neurons in the layer. The activation functions utilized are usually the Signum function or Sigmoid function; the gradient descent method is the most frequently used training optimization algorithm. The specific configuration of BPNN would be set according to the actual dataset and training status.

In order to achieve a high-quality performance of the GNSS receiver in harsh conditions, we delved into the mathematical model and specific implementation process of the navigation receiver clock bias prediction model. Let the GNSS pseudo-range measurement equation, when the number of observable satellites is *M*, be defined as follows [15], in the condition of single-epoch pseudo-range positioning under varying ionosphere delays.

(6) $$\begin{array}{c} \begin{array}{c} \rho_{i}=\left|r-r_{i}\right|+b+\varepsilon_{i}=\\ {\left[{\left(x_{i}-x\right)}^{2}+{\left(y_{i}-y\right)}^{2}+{\left(z_{i}-z\right)}^{2}\right]}^{1/2}+b+\varepsilon_{i} \end{array} \end{array}$$

where $\rho_{i}$ and $\varepsilon_{i}$ represent the pseudo-range measurement value and measurement error, respectively, $r_{i}=\left(x_{i},y_{i},z_{i}\right)$ represents the satellite coordinates, and i means the i-th satellite in a total number of *M*. GNSS receiver coordinates $r=\left(x,y,z\right)$ and clock bias *b* are unknown parameters that need to be solved. In the solving process, Equation (6) is firstly analyzed using a Taylor series expansion at approximate coordinates $\left(x^{0},y^{0},z^{0}\right)$ of the GNSS receiver, omitting terms of second order or higher, to obtain the linearized observation equation [16].

(7) $$\begin{array}{c} L=HX+\nu \end{array}$$

where $L\in R^{m\times 1}$ represents the pseudo-range bias vector, $X\in R^{4\times 1}$ contains the correction amounts $\delta_{x}$, $\delta_{y}$, and $\delta_{z}$ of the GNSS receiver’s approximate coordinates $\left(x^{0},y^{0},z^{0}\right)$ and clock bias parameter b, $H\in R^{M\times 4}$ is the coefficient matrix, and *v* is the error item.

Owing to Equation (7) containing four unknowns, at least four satellites are required for solving. However, in certain adverse conditions where GNSS satellite signals are obstructed, the receiver can only receive signals from three satellites. Under such circumstances, the system has only three observation equations, which is insufficient to solve the four unknown parameters, resulting in the GNSS receiver being unable to function properly.

To address the challenge of GNSS receiver positioning with only three available satellites, this paper incorporates a clock bias prediction model into the observation equation. The three-dimensional positioning capability of the receiver is achieved through the expansion of the observation matrix.

The extended system equation is obtained by replacing the actual measured value b with predictive value $b^{\prime }$; we can derive the following:

(8) $$\begin{array}{c} L^{\prime }=H^{\prime }X^{\prime }+\nu^{\prime } \end{array}$$

In Equation (8), $X^{\prime }\in R^{3\times 1}$ consists of correction value $\delta_{x}$, $\delta_{y}$, and $\delta_{z}$, $L^{\prime }$ and $H^{\prime }$ represent the pseudo-range bias vector and observation matrix after extension, respectively, and $\nu^{\prime }$ is the error item:

(9) $$\begin{array}{c} L^{\prime }=\left[\begin{array}{c} \rho_{1}-\left|r-r_{1}\right|-b^{\prime }\\ \vdots \\ \rho_{M}-\left|r-r_{M}\right|-b^{\prime } \end{array}\right],H^{\prime }=\left[\begin{array}{ccc} l_{1} & m_{1} & n_{1}\\ \vdots & \vdots & \vdots \\ l_{M} & m_{M} & n_{M} \end{array}\right] \end{array}$$

In the formula, the meaning of $\rho_{i}$ and $\left|r-r_{i}\right|$ is the same as that in Equation (6), and $l_{i}$, $m_{i}$, and $n_{i}$ are the direction cosine between the receiver and satellite i:

(10) $$\begin{array}{c} l_{i}=\frac{x_{i}-x^{0}}{\left|r-r_{i}\right|},\;m_{i}=\frac{y_{i}-y^{0}}{\left|r-r_{i}\right|},\;n_{i}=\frac{z_{i}-z^{0}}{\left|r-r_{i}\right|} \end{array}$$

It is evident that, with the help of integration of the clock bias prediction model, the number of unknowns to be resolved has decreased from four to three. Consequently, in scenarios with only three satellites, the GNSS receiver can achieve three-dimensional positioning successfully.

To fulfill the position solving according to Equation (8), matrix $H^{\prime }$ must be invertible. It is required that the GNSS receiver’s location is not collinear with any two satellites. In this way, the vector $X^{\prime }$ and its norm $∥X^{\prime }∥$ are as follows:

(11) $$\begin{array}{c} \left\{\begin{array}{c} X^{\prime }={\left(H^{\prime }\right)}^{-1}L^{\prime }\\ ∥X^{\prime }∥={\left(\delta_{x}^{2}+\delta_{y}^{2}+\delta_{z}^{2}\right)}^{1/2} \end{array}\right. \end{array}$$

The GNSS receiver coordinates can be obtained by multiple iteration. By comparing $∥X^{\prime }∥$ to the given threshold, whether the positioning algorithm converges can be verified. If $∥X^{\prime }∥$ exceeds the threshold, the receiver’s approximate coordinates $\left(x^{0},y^{0},z^{0}\right)$ are modified according to Equation (12).

(12) $$\begin{array}{c} x^{1}=x^{0}+\delta_{x},\;y^{1}=y^{0}+\delta_{y},\;z^{1}=z^{0}+\delta_{z} \end{array}$$

Then, $\left(x^{1},y^{1},z^{1}\right)$ are used as the new approximate coordinates to participate in resolution. When $∥X^{\prime }∥$ drops below the specified threshold, it indicates that the receiver’s positioning accuracy meets the accuracy requirements and the position computation process is finished.

**Figure 2:**
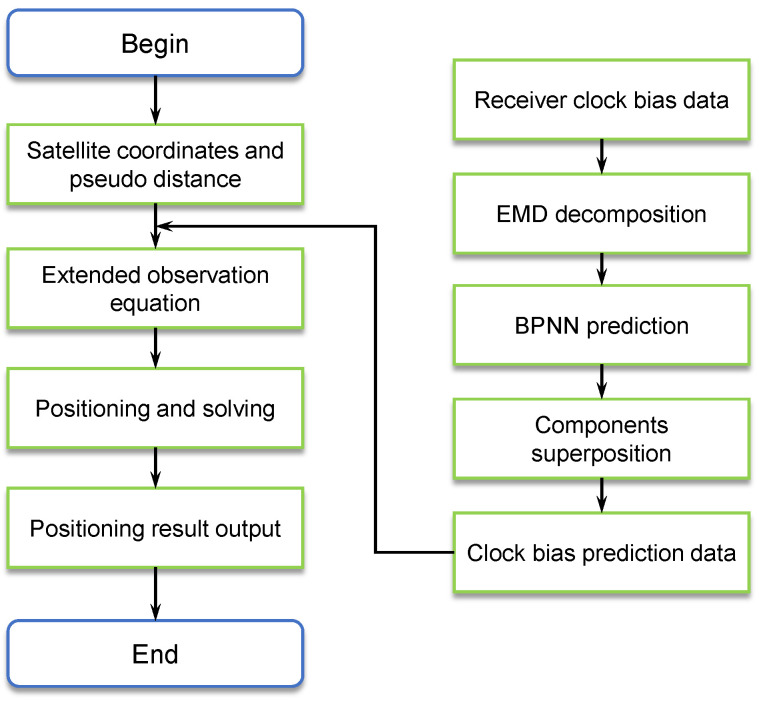
Flow diagram of the clock bias predicting auxiliary positioning method.

Combined with the above analysis, Figure 2 gives a specific process for the GNSS receiver clock bias prediction auxiliary positioning algorithm.

## 3. Experimental Results and Analysis

To investigate the performance of the proposed navigation receiver clock bias prediction auxiliary positioning algorithm, a large number of studies and tests using a GNSS receiver in a real-world environment are carried out. In aggressive environments such as dense urban canyon, the duration during which the GNSS receiver can observe less than four satellites is usually several tens of seconds [3]. To deal with such difficult problems, based on historical clock bias data, the EMD-BPNN clock bias prediction method is utilized to enable the effective positioning in the time period when only three satellites are available. As a consequence, the continuous and uninterrupted high accuracy positioning of the GNSS receiver can be accomplished without additional hardware cost. With the introduction of the proposed clock bias prediction auxiliary positioning algorithm, the GNSS positioning service could keep in good condition during GNSS satellite signal outages. Furthermore, the suggested clock bias prediction algorithm can be used to achieve GNSS positioning augmentation and improve precision compared to the version without clock bias auxiliary prediction, which has also received a lot of attention lately, in the event when more than three GNSS satellites are visible.

**Figure 3:**
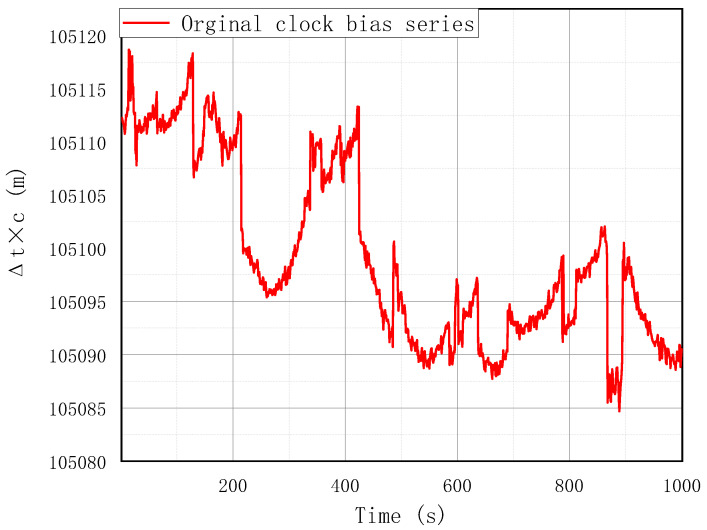
Original acquired clock bias data in field test.

**Figure 4:**
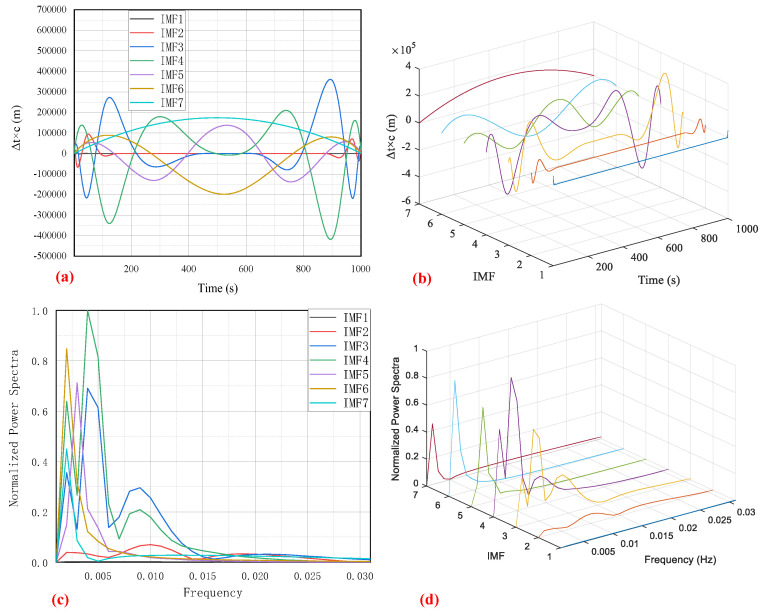
Intrinsic mode IMF components of clock bias signal after EMD decomposition: (**a**) 2D plot in time domain; (**b**) 3D plot in time domain; (**c**) 2D plot in frequency domain; (**d**) 3D plot in frequency domain.

Because of an adequate amount of data available, the training and validation procedure of the EMD-BPNN GNSS receiver clock bias prediction model were implemented on large sets of real acquired data. Before large-scale training, pre-training is carried out to investigate the neural network configuration parameters such as number of nodes in three layers, initial weight and threshold, and so on. After configuration, to avoid converging to a local minimum in the training, differential evolution is chosen rather than the ordinary gradient descent method. The bootstrap method is chosen as the training validation means; the sigmoid function is taken as the activation function. To illustrate the effectiveness and precision of the demonstrated method, a special field test was carried out. The original clock bias data acquired in the field test are shown in Figure 3; the sampling frequency is set at 1 Hz and acquiring time is set at 1000 s; hence, 1000 clock bias data records are used for the model demonstration. A GPS receiver (Njord, from SatLab Geosolutions AB) is used in this experimental demonstration but the proposed method is not limited to it; it also can be applied to other GNSS system such as BeiDou, GALILEO, or GLONASS. There is no intersection between the large training–validation dataset and the filed test acquired data, which prevents overfitting and guarantees the independent effectiveness of the prediction model. In compliance with the aforementioned EMD decomposition procedure, the original clock bias series is decomposed into seven intrinsic mode IMF components which are shown in Figure 4 below.

To elaborate the decomposed IMF components which contain intrinsic mode information on the clock bias signal, the 2D plot and 3D plot of the original acquired signal are shown in the time domain and frequency domain, respectively. The clock bias signal, expressed as Δt×c, is shown in Figure 4a,b as a distance in meters. The normalized power spectra of the clock bias signal after Fast Fourier Transform (FFT) are shown in Figure 4c,d. The high-frequency part of the clock bias signal is negligible for being too weak; therefore, the normalized power spectra are truncated and only the low-frequency signal from 0 to 0.031 Hz is kept. After decomposition, the intrinsic mode IMF components are forecast separately by the BPNN method.

**Figure 5:**
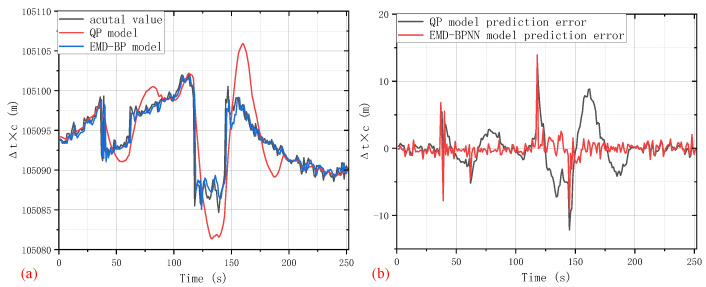
Performance comparison between EMD-BPNN and traditional QP model. (**a**) Clock bias signal prediction result and (**b**) clock bias prediction error.

After forecasting based on the BPNN algorithm, all the predicted values related to different IMF components are superimposed together to obtain the final clock bias prediction. According to the data on the first 75%, the clock bias signal of the last 25% is predicted; moreover, with the introduction of a classical quadratic polynomial clock bias prediction method, the performance of the proposed EMD-BPNN can be fully presented and evaluated. Figure 5a shows a comparison among the EMD-BPNN model predicted clock bias value, QP model predicted clock bias value, and the actual acquired clock bias value. Meanwhile, the clock bias prediction errors of the two algorithms are shown in Figure 5b. It can be seen that the predictive error of our EMD-BPNN model is quite small apart from several abrupt spikes at the 38-th, 39-th, 118-th, and 145-th seconds. Even so, at those special spikes, the clock bias prediction error of the EMD-BPNN method is less than 14 m, and there is no obvious increasing or decreasing trending in prediction error, while additional fluctuations and larger deviation can be observed in the contrastive QP model clock bias prediction error. Obviously, our EMD-BPNN model outperforms the traditional QP model which utilizes a quadratic polynomial to model and forecast the clock bias time series. These results demonstrate convincingly that an effective and practical clock bias prediction is established. After feature extraction using the EMD method, the mapping between the intrinsic characteristics of the IMF mode and clock bias time series future trend is found by BPNN; this combination forecasting approach is proven to be an excellent idea for clock bias predicting research.

**Figure 6:**
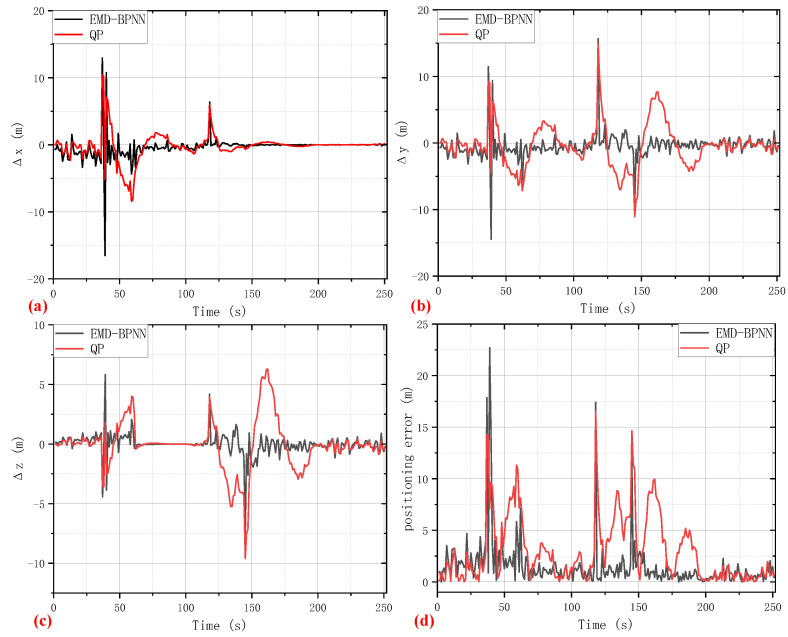
The auxiliary positioning performance of EMD-BPNN and QP models. (**a**) Positioning error in the *x*-axis direction. (**b**) Positioning error in the *y*-axis direction. (**c**) Positioning error in the *z*-axis direction. (**d**) Positioning coordinate error.

With the help of the method mentioned in Section 2, using clock bias data as a virtual satellite, the positioning accuracy based on the clock bias predicted value of the QP and EMD-BPNN models when in harsh conditions is shown in Figure 6. The positioning errors in the *x*-axis, *y*-axis, and *z*-axis directions of Earth-Centered Earth-Fixed (ECEF) coordinates are shown in Figure 6a–c respectively, while Figure 6d illustrates the total positioning coordinate error. Apparently, the performance of the clock bias auxiliary positioning method is intricately linked to the accuracy of clock bias prediction. The more precise the clock bias prediction is, the smaller the positioning error of the GNSS receiver using the auxiliary algorithm. Because of better clock bias predicting performance, the positioning accuracy of the EMD-BPNN model is preferable except for a number of spike points, whereas large amplitude fluctuations appear in the positioning results based on the QP clock bias auxiliary model. It can be observed that, under the adverse condition of the satellite signal being blocked, the maximum positioning error based on the EMD-BPNN clock bias auxiliary model in the *x*, *y*, and *z* directions is about 16 m and the maximum synthesis positioning coordinate error is about 22 m; evidently, at the 37-th, 39-th, 118-th, and 145-th seconds, clock bias prediction errors exceeding 14 m are observed, resulting in maximum positioning errors up to 22 m. For the reason of making a quantitative assessment and comparison of these two algorithms’ performance, a number of widely used evaluation indicators of statistics and machine learning, including Mean Square Error (MSE) and Mean Absolute Error (MAE), are computed and provided in Table 1.

**Figure 7:**
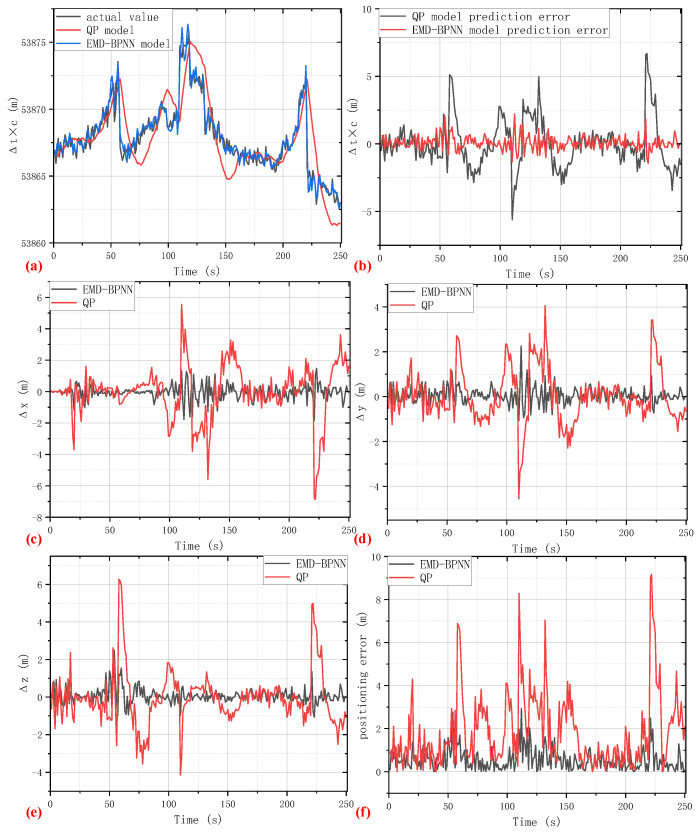
Experiment results using GNSS receiver 2. (**a**) Clock bias signal prediction result. (**b**) Clock bias prediction error. (**c**) Positioning error in the *x*-axis direction. (**d**) Positioning error in the *y*-axis direction. (**e**) Positioning error in the *z*-axis direction. (**f**) Positioning coordinate error.

In order to further demonstrate the efficacy and versatility of the proposed EMD-BPNN algorithm, experimental tests on three other different kinds of GNSS receivers were carried out. The length of the predicting clock bias time series is set at 250 s, which would be sufficient for a majority of GNSS positioning applications such as an Unmanned Aerial Vehicle (UAV) shuttling in a dense urban canyon, a car traveling on a road in densely distributed forest, or a man with a cellphone walking among city tall buildings. Under the abovementioned circumstances, the duration of GNSS satellite signal outages is usually several tens of seconds; by utilizing the EMD-BPNN auxiliary model, the problem of GNSS positioning errors and discontinuity would be resolved. Three different kinds of GNSS receivers were utilized in a supplementary experiment which are denoted as GNSS receiver 2 (Navcom SF-3050), GNSS receiver 3 (Magtempo MGDF-03R), and GNSS receiver 4 (SinoGNSS M900D). Figure 7 illustrates the clock bias prediction and auxiliary positioning results utilizing GNSS receiver 2 with the same experimental setups as the abovementioned field test.

As shown in Figure 7a,b, the occasional large spikes in the clock bias prediction based on the EMD-BPNN method vanish, while the large amplitude fluctuations still exist in the clock bias prediction of the QP model. The positioning errors in the *x*-axis, *y*-axis, and *z*-axis directions, and total positioning coordinate error are shown in Figure 7c–f, severally. Consequently, our EMD-BPNN auxiliary positioning model outperforms the traditional QP model and shows promising positioning accuracy because of a better clock bias prediction performance. The experiment results of GNSS receiver 3 and GNSS receiver 4 are shown in Table 2 in quantitative forms; MSE and MAE are given to clarify the effectiveness of the EMD-BPNN model on different hardware platforms. The characteristics and features of clock bias time series are determined by the crystal oscillator quality of the GNSS receiver and the atomic frequency standard on the GNSS satellite. According to the experimental results, the proposed EMD-BPNN model is proven to have great applicability and versatility; the intrinsic features of the clock bias time series can be found with high efficiency; then, a solid forecasting model is established. After the initial stage of model training, the computational resource cost of the implementation stage including EMD decomposition and forecasting based on established BPNN is quite economical. When the length of the input clock bias time series is 250 s, the time cost of the EMD-BPNN implementation apart from training is 0.17 s on a laptop (Macbook Air M2), which means that the proposed model is appropriate for real-time positioning applications.

All these abovementioned phenomena show that the clock bias auxiliary positioning method can make GNSS receivers maintain accurate and uninterrupted performance unimpeded by satellite signal blocking.

## 4. Conclusions

This paper innovatively proposes a GNSS receiver clock bias time series prediction algorithm which can be applied to harsh environments when the GNSS satellite signal is blocked. The field test results show that our EMD-BPNN approach can make a GNSS receiver maintain normal performance when there are only three satellites in the short term (tens of seconds). According to comparison experimental results, the proposed EMD-BPNN model outperforms the traditional QP model on four different kinds of GNSS receiver platforms. What is more, this method has reference value for future high-accuracy GNSS positioning application for the reason that it does not need additional hardware equipment which improves the device running reliability and reduces cost. The positioning accuracy of a GNSS receiver depends on the performance of the clock bias prediction algorithm. In future work, besides model prediction accuracy promotion, research will also concentrate on how to improve the algorithm interpretability and portability.

## Figures and Tables

**Table 1:** MSE and MAE of the field test’s clock bias prediction error and positioning error using EMD-BPNN and QP models.

		Δx	Δy	Δz	Clock Bias Prediction Value	Positioning Coordinate Error
EMD-BPNN	MSE (m^2^) MAE (m)	2.955 0.5631	4.0676 0.9785	0.8301 0.4358	2.4145 0.7936	7.8532 1.3038
QP	MSE (m^2^) MAE (m)	4.1055 0.9976	10.9676 2.3031	4.40279 1.2367	9.4870 1.9976	19.4760 3.0212

**Table 2:** Experimental performance of EMD-BPNN model on GNSS receiver 3 and GNSS receiver 4.

		GNSS Receiver 3		GNSS Receiver 4	
		Clock Bias Prediction Value	Positioning Coordinate Error	Clock Bias Prediction Value	Positioning Coordinate Error
EMD-BPNN	MSE (m^2^) MAE (m)	1.2264 0.7798	3.4595 1.2284	1.3412 0.7815	3.4772 1.2536
QP	MSE (m^2^) MAE (m )	10.5483 2.3631	23.6136 3.5837	10.4251 2.2523	21.0364 3.3030

## Data Availability

The data presented in this study are available on request from the corresponding author. The data are not publicly available due to privacy or ethical restrictions.
